# Analysis of sectoral participation in the development of Joint External Evaluations

**DOI:** 10.1186/s12889-019-6978-8

**Published:** 2019-05-23

**Authors:** Emily McPhee, Gigi K. Gronvall, Tara Kirk Sell

**Affiliations:** 1 Johns Hopkins Bloomberg School of Public Health, 615 N. Wolfe Street, Baltimore, MD 21205 USA; 2 Johns Hopkins Center for Health Security, 621 East Pratt Street, Suite 210, Baltimore, MD 21202 USA

**Keywords:** Joint external evaluation, Global Health security agenda, International health regulations, Preparedness, Response, National action plan, Multisectoral

## Abstract

**Background:**

The Joint External Evaluation Process (JEE), developed in response to the 2014 Global Health Security Agenda (GHSA), is a voluntary, independent process conducted by a team of external evaluators to assess a country’s public health preparedness capabilities under the 2005 International Health Regulations (IHR) revision. Feedback from the JEE process is intended to aid in the development of national action plans by elucidating weaknesses in current preparedness and response capabilities.

**Methods:**

To identify gaps in sector participation and the development of national action plans in response to public health emergencies, all English-language JEE reports available on March 31, 2018 (*N* = 47) were systematically reviewed to determine sectoral backgrounds of key host country participants.

**Results:**

Overall, strong representation was seen in the health, agriculture, domestic security, and environment sectors, whereas the energy/nuclear and defense sectors were largely under-represented.

**Conclusions:**

While strong participation by more traditional sectors such as health and agriculture is common in the JEE development process, involvement by the defense and energy/nuclear sectors in the JEE process could be increased, potentially improving preparedness and response to widespread public health emergencies.

**Electronic supplementary material:**

The online version of this article (10.1186/s12889-019-6978-8) contains supplementary material, which is available to authorized users.

## Background

Borne in part out of the challenges faced by public health during the 2003 SARS outbreak, the 2005 revision of the International Health Regulations (IHR) guidelines reflected the need to address preparedness in response to public health threats on a global scale [1, 2]. Nearly a decade later, the Global Health Security Agenda (GHSA) convened representatives from a variety of countries, international organizations, and stakeholders to elevate and coordinate response strategies to serious infectious disease threats and address insufficient compliance to the 2005 IHR [3]. The GHSA also elucidated the need for a method by which a country’s capacity for responding to public health threats could be assessed [4, 5]. The Joint External Evaluation (JEE) process, derived as part of the IHR Monitoring and Evaluation Framework, was developed to meet these needs [1, 6]. The JEE is a voluntary, independent process conducted by a team of external peer evaluators to assess a country’s public health preparedness capabilities across 19 technical areas, including response to infectious disease, chemical, radiologic, and nuclear threats [2, 7]. Feedback from the JEE process is intended to aid in the development of a national action plan, targeting weaknesses in current preparedness capacities and fostering increased collaboration between sectors [1, 8].

This focus on multisectoral collaboration in public health preparedness efforts is increasing with the recognition that successful preparation for public health emergencies does not fall solely under the responsibility of the health sector [9]. Rather, this notion emphasizes collaboration across multiple disciplines on the local, national, and global scale in an attempt to foster a more holistic and integrated view of preparedness [6, 10]. Multisectoral involvement in preparedness efforts is not restricted to public entities. Continued functioning of civil society in crisis relies on business continuity plans developed by private entities including non-governmental organizations (NGOs) and community actors to ensure that essential services are ongoing [9]. The GHSA’s 2014 launch highlighted the importance of the connection between human, animal, plant, and environmental health in responding to biological threats [11]. The IHR’s aim to prevent, detect, and rapidly respond to naturally-occurring and deliberate public health threats however, emphasizes the need for an expanded integration of sectors including defense, law enforcement, and intelligence agencies to provide security expertise in preparedness efforts. Though collaboration across different sectors can pose many challenges [12], the implementation of the JEE process provides an opportunity for different sectors to come together and establish a coordinated action plan in the event of a public health emergency.

A standardized scoring process in the JEE allows for a systematic evaluation of country capacities in predetermined technical areas. However, gaps in involvement of institutions by sector across JEE participants may highlight absent sectors in need of inclusion in the JEE process and global health in general. This study provides an analysis of host country participation by sector in the JEE process through an evaluation of institutions that contributed to JEE mission reports in addition to sector backgrounds of evaluation team members. The intent of this research is to help to identify gaps in sector participation that may bias or limit JEE results. Understanding where sectoral gaps lie in the JEE process can better serve to inform the development of a national action plan not only through identifying a country’s technical weaknesses, but also sectors whose involvement will further bolster response capacity to public health threats.

## Methods

To conduct this analysis, we systematically identified and reviewed available JEE mission reports. Reports were obtained from the listing of all English-language JEE reports made available by the World Health Organization (WHO) as of March 31, 2018. Countries with French-only JEE reports that were excluded from this analysis include Cameroon, Chad, Comoros, Guinea, Madagascar, and Mali. Data on key host country participants and institutions and JEE mission team members conducting the evaluation for each respective host country were abstracted from collected documents using a Microsoft Excel-based electronic data collection form.

Key host country participants and institutions were grouped into the following categories based on the participant’s sector and/or the corresponding institution’s purpose: health (including sub-category for food and drug safety, patient care/hospitals, epidemiology/surveillance, laboratory and testing services, nutrition/food security, environmental health, emergency/ambulatory services, zoonotic diseases, and preventative health), agriculture, defense, domestic security, executive/congressional policy, international cooperation, transportation (including sub-category for ports), labor, environment, commerce, communication/media, energy/nuclear, and other (Table 1). Organizations were included in one or more categories if their function in preparedness efforts encompassed multiple sectors. Organizations with unclear sector involvement or functionality underwent further review and were categorized based on their mission statement. Participant organizations identified only through acronyms that could not be identified were placed in the other category.

**Table 1:** Key Host Country Participants and Institutions, Sectors and Definitions

Sector	Definition
Health	Consists of a range of institutions relating directly to healthcare, including Ministries of Health, hospitals, laboratories, and epidemiological surveillance programs
Health (Food and Drug Safety)	Sub-category of Health, including food and drug authorities, foodborne disease prevention, and clinical trials management
Health (Patient Care/Hospitals)	Sub-category of Health, consisting of a variety of organizations relating to patient care including hospitals, clinics, blood transfusion, and immunization services
Health (Epidemiology/ Surveillance)	Sub-category of Health, relating to organizations centered around monitoring of disease prevalence and transmission, including Centers of Disease Control and disease-specific surveillance organizations
Health (Laboratory/ Testing Services)	Sub-category of Health, referring to laboratories in various settings used to detect and confirm infection status
Health (Nutrition/ Food Security)	Sub-category of Health, consisting of organizations ensuring adequate quality and quantity of food consumption by citizens
Health (Environmental Health)	Sub-category of Health, relating to organizations focusing on the environment as it relates to human health
Health (Emergency/ Ambulatory Services)	Sub-category of Health, encompassing organizations centered around emergency/disaster response, including ambulatory services
Health (Zoonotic Disease)	Sub-category of Health, consisting of organizations combatting or researching diseases endemic to both humans and animals
Health (Preventative Health)	Sub-category of Health, relating to organizations involved in disease prevention and health promotion
Agriculture	Encompasses institutions and agencies relating to food production and security including Ministries of Agriculture, animal production and health, fisheries, plant protection, and veterinary services
Defense	Includes Ministries of Defense, host country militaries/armed forces, and weapons protection agencies
Domestic Security	Encompasses a large range of functionalities including law enforcement, justice departments, and emergency and disaster preparedness institutions
Executive/Congressional Policy	Pertains to general legislative bodies within the host country including the Offices of the President and/or Prime Minister and local governments
International Cooperation	Relates to host country institutions engaging in communication with or outreach to other countries, including Departments for International Development, Ministries of Foreign Affairs, and international relations coordination
Transportation	Consists of agencies including Departments of Transportation and Civil Aviation Authorities; includes sub-category for ports, which consists of independent airports and seaports
Labor	Includes Departments and Ministries of Labor
Environment	Includes environmental health and services, wildlife preservation, and climate affairs
Commerce	Encompasses organizations pertaining to the exchange of goods or services, including Ministries of Finance, trade and commerce, and industry
Communication/Media	Includes health communication departments, public telecommunication networks, and national media agencies
Energy/Nuclear	Includes Departments of Energy, radiological protection authorities, atomic energy commissions, and nuclear safety agencies
Other	Encompasses all organizations that did not readily fit into any of the pre-defined categories

While public agencies were divided by primary responsibilities into the aforementioned sectors, private entities were initially divided based on organization type (e.g. business, NGO, state-sanctioned aid, or university). Additional analysis characterized all organizations, both public and private, by sector to determine overall involvement in the JEE and to ascertain how the involvement of private entities served to bolster existing governmental preparedness infrastructure. Sector involvement was defined as having one or more organization representing a particular sector (e.g. heath, agriculture, etc.). Because organizations did not detail the extent or scope of their participation in the JEE process, involvement as a function of the number of organizations within each sector could not be ascertained with confidence. There has been no empirically demonstrated relationship between number of organizations involved in the JEE process and preparedness capacity. Therefore, if at least one organization represented a particular sector, additional numbers of organizations from a particular host country contributing to each sector did not add to the analysis.

Mission team member backgrounds were categorized in a similar manner to host country participants and institutions, separating the organizations that they represent by organization type (e.g. public or private) and subsequently by sector.

Full categorization of key host country participants and institutions and mission team member backgrounds can be found in the Additional file 1.

## Results

### Overall Joint external evaluation representation

**Fig. 1:**
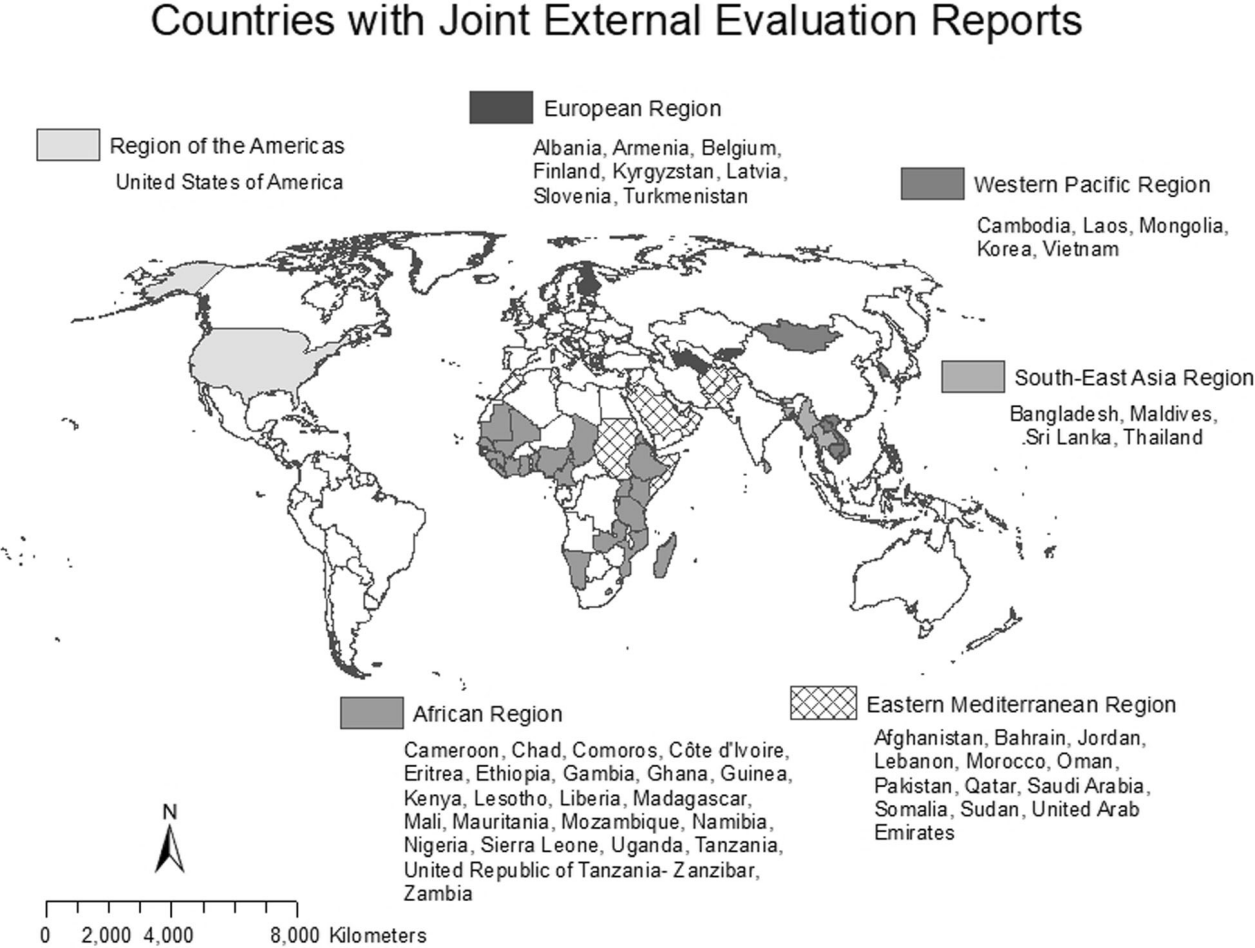
*Map of all countries by WHO region* versus *countries with JEE reports.* Countries with French-language only JEEs included in this map but excluded from analysis include Cameroon, Chad, Comoros, Guinea, Madagascar, and Mali

Of the 193 member states of the WHO, 47 had published English-language JEE reports by March 31, 2018. Regional breakdowns of JEE participants delineate involvement in this process, with the Eastern Mediterranean and African regions providing the most representation in terms of percentage of countries who have gone through the JEE process and the region of the Americas and European region providing the least representation (Fig. 1).

Most countries were well represented in their JEEs by public entities in health (100% of countries), agriculture (95%), domestic security (91%), and environment (86%). Additionally, 72% of countries had representation by the transportation sector, with representation provided by ports rather than governmental departments in 21% of countries. The energy/nuclear (58%), defense (56%), and international cooperation (40%) sectors were less frequently represented in JEEs. Furthermore, within the health sector, the subcategories of patient care/hospitals (65% of countries), epidemiology/surveillance (58%), emergency/ambulatory services (49%), and food safety (49%) were the most well represented, with nutrition/food safety (12%) and zoonotic disease (9%) less frequently represented.

**Fig. 2:**
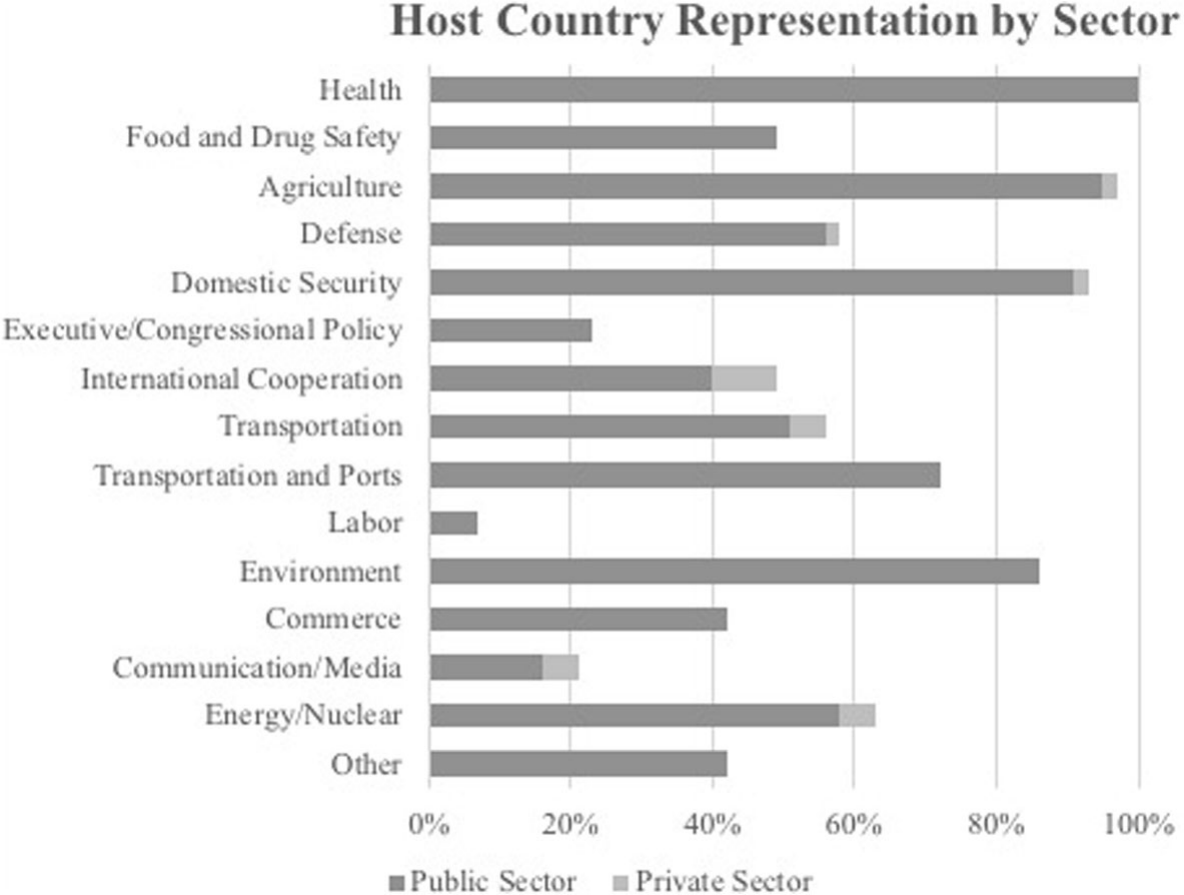
*Categorical Host Country Representation by Sector.* This figure represents the percentage of countries to have included some form of representation for each sector in their Joint External Evaluation. The dark bars represent the percentage of countries that have a public entity representing each category. The light bars represent the percentage of countries whose only form of representation for the particular sector was a private entity

With the exception of privately-owned ports, the addition of involvement by private entities to sector representation in the JEEs did not greatly increase the diversity of representation. Instead, these entities generally bolstered the health and agriculture sectors that were already represented by public entities. (Figure 2) Of the private entities that helped bolster involvement within the health sector, most served to strengthen the epidemiology/surveillance sub-category, with 74% of countries represented overall as compared to 58% with public entities alone. Most involvement from private entities is provided by NGOs, with 58% of countries including an NGO in their JEE, followed by state-sanctioned aid (i.e. aid groups sponsored by a foreign government) (40%), universities (30%), and businesses (16%).

### Regional Joint External Evaluation Representation

Regional differences were seen in the key host country participants and institutions involved in the JEEs. All countries evaluated in the South-East Asia and Western Pacific regions had representation by entities with a national defense mission. In comparison, the defense sector was less often represented in JEEs for countries in the African (50% of countries), Eastern Mediterranean (40%), and European (38%) regions. JEEs in the South-East Asia and Western Pacific regions also showed frequent representation in the international cooperation sector at 75 and 80% respectively. In contrast, organizations involved in international cooperation were less frequently involved in JEEs performed in the African (29% of countries), Eastern Mediterranean (30%), and European regions (25%). One-hundred percent of countries in all regions had representation by the environment sector with the exception of the African and European regions, which had 86 and 50% representation respectively. Additionally, ports contributed significantly to transportation representation in all regions except for the African region.

The Eastern Mediterranean region had the highest representation of business involvement with 60% of countries including a business in their JEE, with the next closest region being African at 36%. Assistance from NGOs was high in the African (86%), South-East Asia (75%), and Eastern Mediterranean (70%) regions. Additionally, the African region had the highest involvement from state-sanctioned aid organizations compared to other regions and over half of all countries in the African, South-East Asia, and European regions had assistance from universities in the JEE process. Regional data from the Region of the Americas is not included as it is only represented by the United States of America.

### Mission team representation

Mission team members primarily represented the health and agriculture sectors, with 100% of JEEs including one or more mission team members with a background in health and 85% with one or more mission team members with a background in agriculture. International cooperation, as represented by mission team members in 23% of JEEs, was the next most represented sector.

All JEEs included one or more mission team member(s) from both an international organization and a domestic organization. Independent consultants were also frequently used as mission team members, serving on the mission team for 43% of all JEEs. Notable consultant backgrounds include communication and advocacy specialists, public health legal advisors, an emergency response operations consultant, risk communication consultants, and a laboratory biosafety and biosecurity and quality management system specialist. Mission team members from businesses and universities were present in 11% of JEEs.

## Discussion

### Overall Joint external evaluation representation

Strong representation in the JEE process by the health (100% of countries), agriculture (95%), and environment (86%) sectors is to be expected, as these sectors are traditionally considered essential to public health preparedness. Health in particular relates to every JEE criterion used to assess a country’s capacity for preparedness from prevention efforts including immunization and antimicrobial resistance, detection efforts including epidemiological surveillance and diagnostic testing, and response efforts including medical countermeasures and the deployment of health personnel. Additionally, the link between health and the agriculture and environment sectors, particularly in the case of pandemic preparedness, is readily apparent, as serious issues related to human health such as zoonotic disease and food safety and security encompass agricultural and environmental health as well. Relatively high involvement of the transportation sector (72%) in JEE participants also is not surprising, as the JEE criterion focused on including points of entry in preparedness measures aims to minimize the risk of disease from crossing borders through bolstering quarantine procedures at national entry points. Gupta and colleauges evaluated JEE scores as a quantitative metric of public health preparedness and response capabilities, illustrating a strong correlation between JEE performance and health outcomes and validating the JEE process as a viable tool in global emergency preparedness-building. [13]

Involvement of security agencies has been recognized as essential in developing effective preparedness plans, particularly in surveillance and response operations [14]. The strong representation of security with 91% of countries including a public agency representing the domestic security sector in their JEE is therefore promising, as the institutions within this sector have the capacity to provide the essential functions of maintaining public safety and order through law enforcement branches and contributing to emergency response through disaster management organizations. Despite this, there are many functions that the military has the capacity to provide that institutions in the domestic security sector do not, including providing logistics support on a mass scale, response to large-scale disasters, and peace-keeping operations [9]. Additionally, emergency situations including pandemics may result in situations with military involvement, particularly if there is civil unrest, large-scale refugee situations, border incursions, and the escalation of conflict [9]. Lower involvement by defense (56% of JEE participants) may reflect a need for increased attention to the public health preparedness contributions of this sector.

The energy/nuclear sector (58% of countries) would have a key role in response to a radiological or nuclear event. Ensuring that preparedness measures for these events are in place is part of the IHR 2005 revision, with a JEE criterion specifically devoted to preparedness in radiological emergencies. Other JEE criteria, such as detection operations, personnel deployment, and risk communication depend on a country’s capacity to have a specific plan in place for these types of events, which likely would involve organizations from the energy/nuclear sector.

Strong participation in the health subcategories of patient care/hospitals (65% of countries), epidemiology/surveillance (58%), and emergency/ambulatory services (49%) is promising, as it indicates the inclusion of these key health areas in public health preparedness activities. Low representation by the nutrition/food security (12%) and zoonotic disease (9%) subcategories may be the result of topic areas being represented by entities in other sectors, including agriculture and environment.

The inclusion of private entities in JEEs around the world, primarily NGOs (58% of JEE participant countries) and state-sanctioned aid organizations (40%), followed by universities (30%) and businesses (16%) is promising. Institutions in the private sector are essential to developing business continuity plans in the event of an emergency to ensure that essential services such as medication distribution and basic utilities continue in civil society. They also are instrumental in helping the government with supply chain vulnerabilities and communication technology system failures such as the internet [9]. Though private entities were involved in the JEE process for many countries, they did not serve to increase the diversity of sector representation, merely bolstering the health and agriculture sectors that already had strong representation by public entities. Because private entities play a multitude of roles contributing to the functioning of civil society, they should be more diversely represented in preparedness efforts.

### Regional Joint external evaluation representation

Countries in the South-East Asia and Western Pacific regions had overall strong multisectoral representation, with 100% of countries in these regions including an organization representing the defense and environment sectors and 75 and 80% including an organization representing international cooperation respectively. These findings align with recent efforts by the Association of Southeast Asian Nations (ASEAN), which includes countries from both the WHO South-East Asia and Western Pacific regions, to bolster multisectoral public health preparedness in the region. This commitment to broadening the scope of sectoral involvement in preparing for public health emergencies has been ongoing since the establishment of the ASEAN Multi-Sector Pandemic Preparedness and Response Work Plan in 2008, which assigned identified key sectors in response efforts, created business continuity plans for times of crisis, and linked pandemic preparedness to the disaster response system [9].

In key sectors where representation was high in the South-East Asia and Western Pacific regions, it was markedly low in the Eastern Mediterranean and African regions (defense, 40 and 50% respectively; international cooperation, 30 and 29% respectively). Additionally, countries in the Eastern Mediterranean and African regions had the most representation by private entities, with 86% of countries in the African region and 70% of countries in the Eastern Mediterranean region including an NGO in their JEE and a relatively high proportion of Eastern Mediterranean countries including a business (60%). Furthermore, the majority of countries in the African region included state-sanctioned aid organizations and universities in their JEE. This trend of comparatively lower involvement by public entities in key sectors and the increased enlistment of assistance by private entities may be due in part to political instability and ongoing crises in these regions. Of the countries listed by the WHO as experiencing Grade 2 and Grade 3 public health emergencies, defined as current situations requiring moderate to substantial emergency support by outside groups, 80% of these are from the Eastern Mediterranean and African Regions [15]. Countries with significant public health and political turmoil in the Eastern Mediterranean region in particular have been cited as having technical and political difficulties in the JEE process [15]. Due to the increased enlistment of aid organizations in emergency response situations in these regions, it is not surprising that a higher level of private entities are included as key partners in the JEE process as they may already play a key role in developing the health capacity and infrastructure for these countries.

Compared to its counterparts, the European region had relatively low representation in key sectors including environment (50% of countries), defense (38%), and international cooperation (25%). This is surprising, given the region’s commitment to the European Action Plan to Improve Public Health Preparedness and Response, which emphasizes linking emergency preparedness networks with health systems and improving collaboration between sectors [16]. Our results indicate that the European region may have a different approach to public health preparedness, prioritizing different sectors to include in emergency response plans. Additionally, the lack of representation across sectors may be influenced by the fact that only 15% of WHO European region member states have participated in the JEE process to date, with the majority of these countries concentrated in the eastern region of Europe.

### Mission team representation

While host countries overall have good multisectoral representation in their JEEs, members of the mission teams are overwhelmingly from the health and agriculture sectors. In contrast, the standardized process of mission team staffing emphasizes organizational and background diversity [5]. Though a need for strong multisectoral representation in developing preparedness action plans has been demonstrated to be beneficial, the importance of mission team members from diverse backgrounds is not clear. The ability to evaluate a country’s capacity to respond to the predetermined evaluation criteria in the JEE process may not be dependent on multisectoral representation by peer evaluators.

### Limitations

A total of 6 JEE reports, not published in English, were excluded from the analysis to avoid misclassification of key participants into relevant sectoral categories due to translation errors, limiting the representativeness of this study. This analysis relied on host country lists of key participants in JEE development. The extent to which key participants are engaged in the JEE process was not delineated by Member States and therefore could not be addressed in this analysis. Additionally, because sector involvement was defined as a country having one or more organizations representing a particular sector, our results do not distinguish between countries with more or fewer participating organizations. Our analysis does not intend to convey a country or region’s level of preparedness within any particular sector, but rather to determine engagement across multiple sectors and identify overall gaps in the hopes of informing which sectors need to be bolstered to facilitate a more integrated approach to preparedness.

## Conclusions

While public health preparedness has long been viewed primarily as the responsibility of the health sector, countries in all regions of the world are illustrating their capacity to engage multiple sectors and private entities, both international and domestic, into their preparedness plans through the JEE process. Strong representation overall by the health, agriculture, and environment sectors in the JEE process to date, while necessary, is not sufficient in mounting an effective response to a widespread public health emergency. A strong multisectoral approach to public health preparedness as defined by the 2005 IHR revision and the criteria set forth in the JEE process will require increased involvement of the defense and energy/nuclear sectors in countries around the world moving forward. The JEE process however is iterative and as countries stay committed to these external evaluations, multisectoral preparedness capacity should only increase over time. Through identifying sectoral gaps in the JEE process in this analysis, we hope to facilitate adherence to the revised 2005 IHR guidelines by countries around the world through prioritizing the inclusion of traditionally underrepresented sectors such as defense and energy/nuclear in public health preparedness planning.

## Additional file

Additional file 1: Categorization of key host country participants and institutions and mission meam member backgrounds. (XLSX 153 kb)

## Abbreviations

ASEAN: Association of Southeast Asian Nations
GHSA: Global Health Security Agenda
IHR: International Health Regulations
JEE: Joint External Evaluation
NGO: Non- governmental organization
WHO: World Health Organization
